# The Potential Role of Nasal Cytology in Respiratory Diseases: Clinical Research and Future Perspectives

**DOI:** 10.3390/jcm14030884

**Published:** 2025-01-29

**Authors:** Giuseppina Marcuccio, Giuseppina Raffio, Pasquale Ambrosino, Claudio Candia, Elena Cantone, Aikaterini Detoraki, Mauro Maniscalco

**Affiliations:** 1 Istituti Clinici Scientifici Maugeri IRCCS, Pulmonary Rehabilitation Unit of Telese Terme Institute, 82037 Telese Terme, Italy; giuseppina.marcuccio@icsmaugeri.it (G.M.); giuseppina.raffio@icsmaugeri.it (G.R.); 2 Istituti Clinici Scientifici Maugeri IRCCS, Scientific Directorate of Telese Terme Institute, 82037 Telese Terme, Italy; pasquale.ambrosino@icsmaugeri.it; 3 Department of Clinical Medicine and Surgery, Federico II University, 80131 Naples, Italy; claudio.candia@icsmaugeri.it; 4 Department of Neurosciences, Reproductive Sciences and Odontostomatology, Federico II University, 80131 Naples, Italy; elena.cantone@unina.it; 5 Department of Internal Medicine and Clinical Complexities, A.O.U. Federico II, 80131 Naples, Italy

**Keywords:** nasal cytology, asthma, biomarkers, outcome, rehabilitation, disability, chronic rhinitis

## Abstract

Nasal cytology is a non-invasive, affordable, and easily executable technique commonly used in research to study rhinitis and, to a lesser extent, chronic rhinosinusitis. It is particularly useful for the differential diagnosis of non-allergic rhinitis and for phenotyping chronic rhinosinusitis. Allergic rhinitis, asthma, and aspirin intolerance are frequent comorbidities of chronic rhinosinusitis. A diagnostic system has been proposed to assess the severity of chronic rhinosinusitis (clinical-cytological grading), incorporating nasal cytology and comorbidity observation. This score correlates with the recurrence risk of chronic rhinosinusitis with nasal polyposis. Specifically, a higher grade is often linked to asthma, aspirin intolerance, a recurrent disease requiring surgery, and a mixed cell phenotype (eosinophilic and mast cell). Although nasal cytology has been shown to be able to replace bronchial analysis with acceptable precision due to its technical characteristics, its use in diseases affecting both upper and lower airways remains limited. The main limitation of this technique is its lack of standardization, which currently hinders its widespread clinical adoption despite its increasing familiarity among allergists and otolaryngologists. In the context of the unitary airways hypothesis, nasal cytology could also provide valuable insights for managing lower airway diseases like chronic obstructive pulmonary disease and obstructive sleep apnea syndrome, which significantly impact quality of life and healthcare costs. This review aims to provide an overview of nasal cytology, highlighting its limitations and potential applications in chronic respiratory diseases.

## 1. Introduction

The human nose represents the entrance gate for inhaled air and, therefore, the first anatomical and functional system that provides its purification, warming, and humidification in order to “direct” it toward the lower airways and make it “suitable” to reach the pulmonary alveoli, where gas exchanges occur [1]. This complex set of actions is made possible by the respiratory nasal epithelium, whose ciliated and mucous cells cover the entire nasal cavity, except for the vestibular and roof areas, which instead have a keratinized stratified squamous epithelium and an olfactory epithelium, respectively [2].

Such nasal respiratory epithelium is similar to the one that lines the lower airways, and both also participate in immune defense mechanisms that can act synchronously in these two anatomical compartments against both pathogenic and non-pathogenic external agents, as well as proteins that act as allergens. Through complex molecular pathways, this protective system is responsible for the influx of inflammatory cells (neutrophils, eosinophils, basophils, mast cells, lymphocytes, and plasma cells), which maintain the state of immune inflammation [3].

Common cytological methods that study inflammatory processes of the lower airways include induced sputum analysis, bronchoalveolar lavage, and bronchial brushing; the first is a non-invasive technique, while the others are both invasive [4,5,6]. Similar and “parallel” techniques for studying nasosinusal pathologies, usable in clinical practice and thus recommended by the most common guidelines, have yet to be recognized and validated. Particularly, the latest European Position Paper on Rhinosinusitis and Nasal Polyps [7], published in 2020, did not recommend performing either biopsy or other kinds of sampling at the first diagnosis of chronic rhinitis, although the steering committee did not achieve a unanimous consent on whether such methods should be applied in case of treatment failure. Nonetheless, the same document advocated for a better evaluation of cytological features in patients with chronic rhinosinusitis, particularly eosinophilia, given its impact on prognosis and treatment [8].

In this regard, nasal cytological examination through scraping or brushing of the nasal mucosa has been added to various techniques for studying rhinitis and rhinosinusitis (e.g., fiberoptic endoscopy, immunohistochemistry on histological samples), aiming to observe and analyze the cellular components present on the mucosal surface of the nasal respiratory epithelium. In particular, nasal cytology has been proposed as a versatile and non-invasive tool for the diagnosis and monitoring of upper airway diseases [2].

In light of the above, the aim of the current narrative review is to present the latest evidence concerning the feasibility and clinical utility of nasal cytology as well as its potential novel applications in the field of respiratory diseases.

## 2. Techniques for Nasal Cellular Sampling: An Overview

Multiple techniques can be used to obtain nasal cellular samples, including nasal lavage, nasal swabs, direct aspiration through microcannulas, nasal brushing, nasal scraping, and, finally, nasal biopsy, which allows for a complete histological evaluation [9]. These techniques differ in outcome, invasiveness, and execution complexity.

Specifically, they range from the non-invasiveness of nasal lavage techniques and nasal scraping to the invasiveness of nasal biopsy; brushing is considered a minimally invasive technique [9]. Regarding execution complexity, nasal scraping and brushing are certainly the easiest to perform [9]. Compared to brushing, nasal scraping allows for greater reproducibility associated with better optimization in observing epithelial cells, as shown in Table 1, although studies evaluating the accuracy of sampling techniques are still limited [10,11].

In this regard, a recent clinical study conducted on 215 patients with reported nasal symptoms estimated the sensitivity and specificity of nasal cytological analysis to be 100% and 49.6%, respectively, with an overall accuracy estimate of 69.5%, due to its high ability to identify true-positive NAR cases and its low capacity to recognize false-negatives [13]. A similar study conducted on 48 patients with chronic rhinosinusitis and 20 healthy subjects compares the cytological technique using brushing on the inferior turbinate, processed in liquid phase, with histological biopsy of the same anatomical site. This technique seems to have high sensitivity and specificity, respectively 94.1% and 76.9%, with a positive predictive value of 84.2% for nasal inflammatory processes [14].

The examination of nasal cells (rhinocytogram) through nasal scraping is currently the most used technique in clinical research in the field of rhinoallergology to deepen the study of rhinitis and rhinosinusitis, allowing for a “snapshot” of the cellularity underlying a more complex clinical picture [2]. As early as 1988, Meltzer and colleagues proposed its use in clinical practice to provide more precise diagnostic information in inflammatory diseases of the nasal mucosa and paranasal sinuses, which would be useful for therapeutic purposes [12]. Specifically, the authors, considering that the management of patients with naso-sinusal disease requires more detailed tests than clinical history, classical physical examination, and allergy tests, highlighted the effectiveness of nasal cytological analysis, comparing its observational potential with other methods (cytological analysis of secretions and histological biopsy), although acknowledging its limitations (Table 1) [12]. In the years following Meltzer’s hypothesis, it was not until 2006 that the concept of nasal cytology was introduced as a systematized and in-depth technique useful for diagnosing non-allergic rhinitis (non-allergic rhinitis, NAR) and phenotyping chronic rhinosinusitis (chronic rhinosinusitis, CRS) with polyps (CRSwNP) and without polyps (CRSsNP) [15,16].

In the last 15 years, the hypothesis of unified airways, understood as a single entity from an anatomical and functional perspective, has been applied to several chronic inflammatory diseases that manifest synchronously in both the upper and lower respiratory tracts, such as CRSwNP and CRSsNP, asthma, allergic rhinitis (AR), and non-allergic rhinitis (NAR), also known as cellular rhinitis [9]. These include non-allergic neutrophilic rhinitis (NARNE), non-allergic eosinophilic rhinitis (NARES), non-allergic mastocytic rhinitis (NARMA), and non-allergic eosinophilic and mastocytic rhinitis (NARESMA) [17,18]. Similarly, as we will see below, the fields of scientific application of nasal cytology could be extended to other chronic inflammatory diseases of the respiratory tree for a more comprehensive interdisciplinary management of the disease process as a whole [9].

## 3. Nasal Cytology in Practice

### 3.1. Performing Nasal Cytology

**Figure 1:**
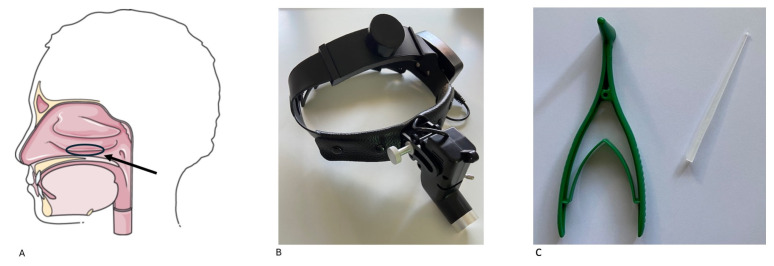
(**A**): Preferential site for scraping (medial surface of middle third of the lower turbinate). (**B**,**C**): A front light cap and sterile curette were used for sampling.

In order to study the cellular content of the nasal respiratory mucosa and its superficial secretions, a nasal scraping is usually performed. With this technique, a sample from the medial surface of the middle third of the lower turbinate is collected under visual control using a front light cap, a sterile curette or a sterile cotton swab (cotton tip), especially in children and in those cases where performing an anterior rhinoscopy is not possible due to lack of equipment and/or experience [19,20] (Figure 1).

In both cases, this is a non-bioptic procedure but a cytological sample of the mucosal surface through exfoliation: a simple and completely painless practice that does not require any local anesthesia. Once the sample is taken, it is transferred onto a slide using a smear and fixed in the air or with 95% ethyl alcohol [21]. May-Grunwald-Giemsa or differential staining allows the identification of all cellular components (neutrophils, eosinophils, lymphocytes, and mast cells), bacteria, and fungal spores/hyphae using two different dyes, which highlight the cell components differently depending on the pH value. May-Grunwald is a mixture of two dyes: methylene blue eosinate, which stains the nuclei in blue and the basophilic cytoplasm in a red-pink shade. Giemsa is a complex mixture of methylene blue chloride, methylene blue eosinate, and azure II eosinate, which enhances the intensity of nuclear staining and selectively highlights cellular elements. The first step, or primary staining, involves using pure May-Grunwald dye in an amount sufficient to cover the entire slide. During this three-minute process, cells are fixed through dehydration mediated by the methanol present in the dye. The slide is then immersed in a solution of 50% May-Grunwald and 50% distilled water for 6 min, followed by washing the slide with distilled water to remove excess dye. The second step, or secondary staining, involves using Giemsa dye diluted 1:10 with distilled water for 30 min, which is useful for differentiating cells. After this time, the slide is washed with spring water and left to dry [2,22]. An alternative to the staining described above is a rapid variant called Fast May-Grunwald-Giemsa (MGG Quick stain, Bio-Optica^®^, Milano, Italy) [19]. This method involves three reagents: reagent A (Methanol), reagent B (Eosin in phosphate buffer), and reagent C (Tiazine dye in phosphate buffer). The procedure using the fast technique consists of immersing the slide five times for one second in reagents A, B, and C. Finally, wash with spring water and allow to dry in a vertical position. The color pattern is strongly influenced by the pH of the washing water and the dilution buffer; the intensity of the staining may vary depending on the differentiation times.

**Figure 2:**
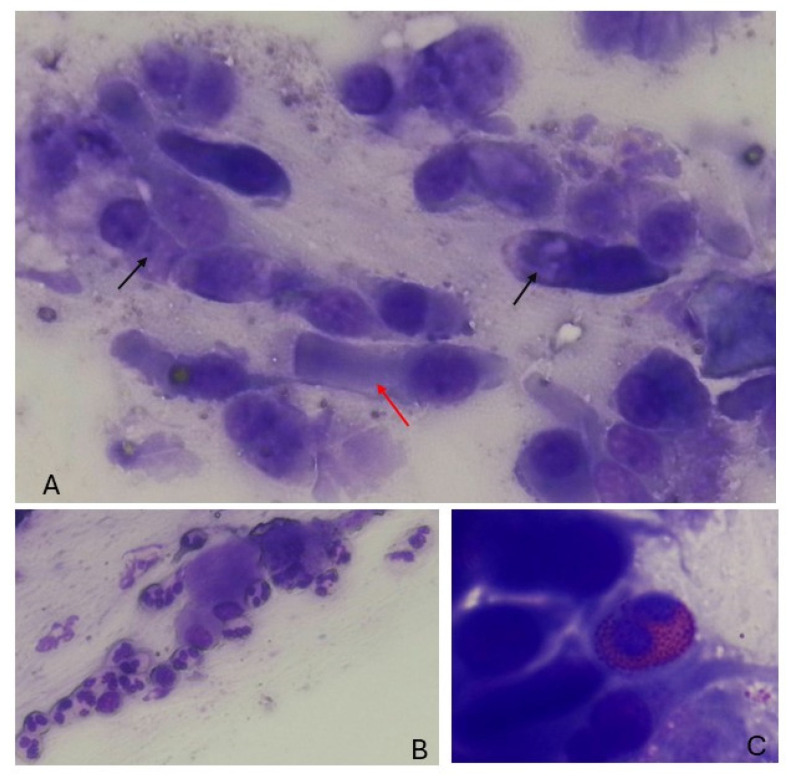
Nasal scraping samples. (**A**): ciliary cells (red arrow) and mucous cells (black arrow). (**B**): neutrophils. (**C**): eosinophil cell.

Microscopic observation is performed using a 100× objective (total magnification of 1000×) with immersion oil [19] (Figure 2). Observation of at least 50 microscopic fields is required to obtain a valid cell count for diagnostic purposes [12] (Table 1).

### 3.2. What Information Can Be Gained Through Nasal Cytology?

The percentage of individual cells, as well as any bacteria and spores, together allows for differential diagnosis between various forms of rhinitis, optimizing treatment [12,19]. In this way, it is possible to observe the specific phenotype of what was previously understood as vasomotor rhinitis in a broad sense through the identification of the cells present and to recognize mixed overlapping forms of difficult therapeutic control, according to guidelines, which may be responsible for Severe Chronic Upper Airway Diseases (SCUADs) [2,23]. In this regard, Bousquet et al. established a list of priorities in studying SCUADs, with the definition of phenotypes of chronic nasal inflammation at the top, followed by association with comorbidities such as asthma [24], emphasizing the need for in-depth and parallel study of the upper and lower airways.

In order to identify non-allergic forms overlapping with pollen AR, in Italy, it is recommended that the nasal cytological procedure be performed in November when exposure to common aeroallergens is lower. For NAR, due to dust mite sensitization, it is recommended that the sampling be performed during summer [25].

Microscopic observation of a nasal scraping sample also reveals the presence of ’infective spots’, which are the equivalent of the biofilm observed in ultramicroscopy [26]. This represents a solid biochemical structure based on polysaccharides produced by bacteria and fungi, which, due to their structural characteristics, are particularly protected from the host’s immune defenses and antimicrobial molecules [27]. The bacteria within the biofilm, compared to those in the fluids and tissues in planktonic form, have the characteristic of transcribing DNA synchronously as if they were part of a multicellular organism capable of sending planktonic bacteria at a distance [28]. Research studies have observed that the infective spot, seen in microscopic examination of nasal scraping samples, as a ’cyan spot’, is positive for staining with periodic acid-Schiff, confirming its polysaccharide structure typical of biofilm [26]. A moderate body of evidence has underscored the clinical relevance of fungal infections in chronic rhinosinusitis [29], and a recent report has shown that the local aspiration of nasal secretions is able to provide suitable material for the diagnosis of eosinophilic fungal rhinosinusitis [30].

Nasal scraping is also used in research to study the motility of ciliated cells. In this case, the collected material is placed at the center of the slide with a few drops of physiological solution at 36 °C and covered with a coverslip. Microscopic observation with a phase contrast lens system and maximum immersion magnification (×1000) allows for the recording of ciliary beat frequency (CBF), classified as present (3–4 beats per second, beats/s), suboptimal (1–2 beats/s), or absent [9,31]. This last application could provide useful information for identifying primary and secondary dyskinesias that may be associated with chronic rhinosinusitis and lower airway diseases such as bronchiectasis, warranting further diagnostic investigation [32].

In summary, the main clinical applications of the nasal cytological study technique are included in the diagnosis of various forms of rhinitis (Table 2) and in the phenotyping of CRS, as previously mentioned. In particular, within the context of chronic respiratory diseases, the literature acknowledges that this methodology provides, together with careful clinical and instrumental evaluation, a diagnostic-therapeutic framework in CRSwNP through the analysis of clinical-cytological grading (CCG) [9].

**Figure 3:**
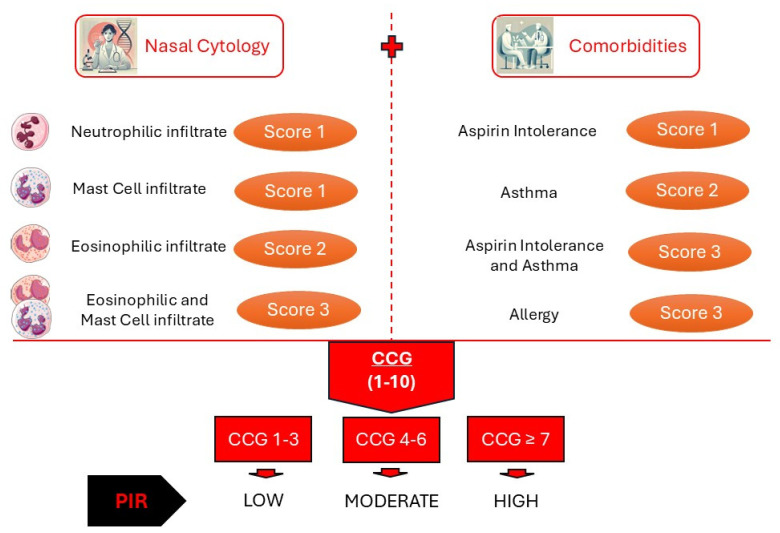
Clinical-cytological grading (CCG) algorithm for Prognostic Index of Relapse (PIR) assessment in Chronic Rhinosinusitis with Nasal Polyps (CRSwNP) [33].

CCG is assigned based on nasal cellularity and comorbidities associated with CRSwNP [33] (Figure 3). The resulting score reflects the severity of the disease, providing the prognostic index of relapse (PIR) for a highly recurrent and disabling condition. Specifically, the presence of neutrophils or mast cells is assigned a score of 1, the presence of eosinophils a score of 2, and the presence of both eosinophils and mast cells a score of 3. Similarly, a positive history of aspirin intolerance is assigned a score of 1, asthma a score of 2, and allergy a score of 3. In this way, the CCG ranges from 1 to 10, allowing for the determination of disease severity from Low for CCG between 1 and 3, Moderate for CCG between 4 and 6 and High for CCG ≥ 7 [34].

### 3.3. Main Limitations

Although nasal cytology is a widely described and regulated technique [35] and despite that it allows nasal cell sampling through a non-invasive approach in the diagnostic process of airway inflammatory diseases and in therapeutic follow-up, it remains underused in clinical practice, as it is still not codified in the guidelines for the diagnosis and treatment of rhinitis and rhinosinusitis.

Nasal cytology is, in fact, burdened by intrinsic limitations, as it is a semiquantitative technique that is prone to operational errors and interpretative mistakes. In its three procedural phases—sampling (nasal scraping), processing (fixation and staining), and microscopic observation (diagnostic reading)—there is currently no consensus in the literature regarding its standardization despite its extensive use in scientific research. In this regard, some authors have compared two processing techniques, using the smear method on a slide or the classical method and centrifugation with prior dilution of the sample, on 105 patients with AR, NAR, dry rhinitis, CRSwNP, and CRSsNP. The results of this comparison conclude that there is no difference between the two techniques, although centrifugation provides a higher-quality sample on the slide with no overlapping cellular elements, thus facilitating reading [36].

The technique for nasal cytology is also time-consuming because it requires dedicated time, especially during the microscopic observation phase. In response to this need, a digitalized reading system through scanning cellular preparations, compatible with the latest generation optical microscopes, has made it possible to develop deep-learning-based software that can assist in the reading phase, as previously described by some authors [37,38].

Finally, in addition to the aforementioned limitations, this technique is operator-dependent and requires highly qualified personnel and appropriate equipment.

## 4. Applications of Nasal Cytology in Clinical Research

In a large retrospective clinical study conducted on 791 patients with CRSwNP, 50.8% of the patients had AR, 30.8% had concomitant asthma, and 24.8% had aspirin-exacerbated respiratory disease (AERD). Similarly, 11% of the patients had a neutrophilic cytological phenotype, 46.3% had an eosinophilic phenotype, 14.2% had a mastocytic phenotype, and 27.9% had a mixed phenotype. A follow-up analysis of these patients concluded that a high CCG was more frequently associated with asthma, AERD, a history of recurrences requiring functional endoscopic nasal surgery, and the mixed phenotype. In contrast, a low CCG seemed to be associated with the absence of comorbidities, recurrences, and the neutrophilic phenotype [39,40]. In this way, nasal cytological analysis and the presence of specific comorbidities provide the CCG, defining the severity degree of CRSwNP, which is closely related to the PIR, in order to provide personalized treatment [41]. This is an observational study that lays the groundwork for future research protocols aimed at analyzing the effectiveness and accuracy of this score in CRSwNP.

The epidemiological data described in the previous study regarding the presence of asthma in patients with CRSwNP aligns with findings from other studies [42]. Additionally, it is estimated that between 57.1% and 62% of these patients have severe asthma, suggesting that CRSwNP may represent a risk factor for severe asthma with poor therapeutic control [43]. Some authors also suggest that these patients have a high percentage of eosinophils in their sputum and reduced pulmonary functional capacities compared to patients with CRSsNP [44,45].

In the context of comorbidities often associated with rhinitis and CRS, the literature shows a relationship between NARNE and gastroesophageal reflux disease (GERD): a recent clinical study demonstrates that patients with a cytological diagnosis of NARNE have a higher acid exposure time (AET) and a higher number of gastroesophageal reflux episodes compared to patients with other NAR forms [46].

According to some authors, studying nasal cells could even serve as a surrogate for studying bronchial cells [47]. In this regard, a study on airway epithelial cell cultures from bronchial and nasal regions showed that they have the same response to inflammatory stimuli, releasing interleukin (IL)-1β and tumor necrosis factor (TNF)-α, which in turn induce the production of a series of pro-inflammatory molecules such as IL-8, IL-6, and matrix metalloproteinase (MMP)-9 [47]. The main limitation of this study lies in the in vitro cell model, which obviously does not include the complex characteristics of in vivo epithelium, given the absence of structures in the extracellular matrix, and thus, further investigation is required. Nevertheless, it remains relevant that nasal epithelial cell cultures could serve as an accessible surrogate for studying lower airway diseases.

However, the use of nasal cytology for studying many inflammatory diseases of the lower airways remains somewhat limited and requires further exploration.

## 5. Future Perspectives

To date, CRSwNP has been recognized as a relevant comorbidity in patients suffering from severe asthma [48], and some biological drugs targeting specific pathways involved in the pathogenesis of both diseases have been licensed [49], with highly positive results deriving from trials and real-life evidence [50]. However, the identification of type 2 high inflammation biomarkers in CRSwNP is still burdened by a high degree of uncertainty and lack of universal validation. In this context, nasal cytology holds the promise to provide practical and clinically relevant information related to the capability of identifying the degree of local eosinophilic inflammation [51].

In addition to bronchial asthma, other chronic respiratory diseases of the lower airways that deserve further exploration include chronic obstructive pulmonary disease (COPD) and obstructive sleep apnea (OSA). These are complex clinical conditions that negatively impact many socio-health determinants, such as quality of life and medical and rehabilitative healthcare costs [52,53].

Growing scientific evidence suggests that COPD is a disease not confined to the lower airways, as a percentage of cases ranging from 75% to 88% report nasosinusal symptoms [54,55]. Although an association between CRS and COPD is known in the literature [49], the comparative study of nasal and bronchial cellularity remains an area to explore [52]. A prospective cohort study showed that in COPD, there is a relationship between bacterial colonization of the lower airways and similar colonization in the nasal passages, with the possibility of identifying in the post-nasal drip a potential pathogenic mechanism for the spread of infectious inflammation. This finding seems to be associated with a synchronous increase in IL-8 (a neutrophil chemoattractant) in both the upper (nasal mucosa) and lower airways [56]

It would be appropriate to evaluate the cytological characteristics of rhinitis and rhinosinusitis associated with this lower airway disease, not only to deepen the clinical understanding but especially to identify a personalized therapeutic approach, possibly in a multidisciplinary and multidimensional context [57].

Similarly, the association between rhinitis and OSA allows for the identification of a potentially responsive genesis for targeted pharmacological treatments. In this regard, a study conducted on a pediatric population shows that while AR is the basis for habitual snoring, OSA seems to be mainly associated with NAR [58]. Similarly, some have reported a prevalence of AR and NAR in adult OSA patients of 27.1% and 28.7%, respectively, without a direct association with the severity of rhinitis [59]. In contrast, other authors have reported that the prevalence of OSA in adult patients with NAR and AR is 83% and 36%, respectively [60]. A community study conducted on a population with sleep-disordered breathing shows that 69.8% of patients have NAR, with a prevalence more than double that of the general population [61]. Given these findings, it would be appropriate to investigate the forms of NAR implicated in OSA, as this would provide insights into the molecular pathogenesis of the inflammatory process underlying this association and appropriate treatment.

Although the concept of unified airways has gained widespread recognition in recent years, many complex pathological conditions remain underexplored, where a careful study of nasal cells could provide new therapeutic insights for better disease control.

## 6. Conclusions

In conclusion, knowledge of nasal cellularity represents a useful tool in the study of upper airway inflammations, allowing, along with a careful clinical evaluation, the differential diagnosis of various forms of rhinitis, particularly all forms of NAR, which are not otherwise easily identifiable. The methodology of nasal cytology evaluation requires further exploration, especially in the reading phase, where the application of new artificial intelligence techniques could allow for its standardization. The importance of nasal cellular analysis in the genotyping of chronic nasosinusal inflammatory diseases could be further emphasized within the context of the unified airway theory. Indeed, besides asthma, which is extensively studied in CRS management, according to the scientific literature, other inflammatory diseases of the lower airways, such as COPD and OSA, would require a thorough evaluation of potential upper airway inflammation.

## Figures and Tables

**Table 1:** Comparison between three principal sampling methods. Modified from reference [12].

Cellular Elements and Cell/Tissue Sampling Techniques			
Cells/Tissue	Secretions	Scraping	Biopsy
Ciliate Cells	no	yes	yes
Mucoid Cells	no	yes	yes
Eosinophils	yes	yes	yes
Basophils	yes	yes	yes
Mast cells	rare	yes	yes
Neutrophils	yes	yes	yes
Submucosa	no	no	yes
Bacterial Artifacts	frequent	infrequent	infrequent

**Table 2:** Differential diagnosis based on microscopic field observation. Adapted from reference [12] Abbreviations: AR, allergic rhinitis; NARES, non-allergic eosinophilic rhinitis; NARESMA, non-allergic eosinophilic and mastocytic rhinitis; NARNE, non-allergic neutrophilic rhinitis.

	Cellular Elements				Fungal Spore
	Eosinophils	Mast cells	Neutrophils	Bacteria	
Healthy subjects	0	0	0–1+	0	0
AR	2+−/4+	2+−/4+	2+−/4+	0	0
NARES	2+−/4+	0	variable	0	0
NARESMA	2+−/4+	2+−/4+	variable	0	0
NARNE	0	0	3+−/4+	0	0
Common Cold	0	0	1+−/4+	0	0
Bacterial infective rhinitis	0/1+	0	3+/4+	3+/4+	0
Fungal rhinits	0	0	variable	0	2+/4+
Atrophic rhinitis	0	0	variable	0	0

## Data Availability

Not applicable.
